# Peroxisome Proliferator-Activated Receptor **α** Plays an Important Role in the Expression of Monocyte Chemoattractant Protein-1 and Neointimal Hyperplasia after Vascular Injury

**DOI:** 10.1155/2012/970525

**Published:** 2012-06-28

**Authors:** Yu Peng, Qiang Li, Lu Zhang, Ming Bai, Zheng Zhang

**Affiliations:** Department of Cardiovascular Medicine, The First Hospital of Lanzhou University, Lanzhou, Gansu 730000, China

## Abstract

Peroxisome proliferator-activated receptor **α** is a member of the nuclear receptor superfamily. It modulates smooth muscle cell proliferation and inflammatory cytokines in vitro. In this study, we tested the hypothesis that PPAR**α** would decrease the expression of monocyte chemoattractant protein-1 and tissue factor, and inhibit neointimal formation in a murine double carotid artery injury model. Carotid artery injury was performed in the PPAR**α** knockout and wild type (WT) mice, treated and untreated with Wy14643, a PPAR**α** activator. Up-regulated MCP-1 and TF expression and more neointimal formation were observed in the PPAR**α** ^−/−^ mice compared with WT mice. The activation of PPAR**α** resulted in further decreased neointimal formation. Our data further suggest that the decrease in neointimal formation is due to down-regulation of MCP-1 by PPAR**α** resulting in decreased leukocyte infiltration and TF expression.

## 1. Introduction

Studies in animal models and clinical studies have demonstrated a clear link between inflammation and vascular response after injury [1–3]. This link between inflammation and vascular response highlights the potential therapeutic application of anti-inflammatory compounds to inhibit restenosis occurring after vascular injury. Peroxisome proliferator-activated receptor (PPAR*α*) is a member of the nuclear receptor superfamily of ligand-dependent transcription factors. PPAR*α*, as well as the other members of the PPAR family, PPAR*β* and PPAR*γ*, positively regulates gene expression by binding to PPAR response elements in target genes as a heterodimer with retinoid X receptors (RXRs) followed by the recruitment of coactivators and consequent transcription of the target genes [4]. PPAR*α* is expressed in vascular cells including endothelial cells, monocyte/macrophages, and VSMCs [5]. PPAR*α* can be activated by hypolipidemic, eicosanoids, or polyunsaturated fatty acids [6]. It has been shown that its activity modulates smooth muscle cell proliferation and inflammatory cytokine expression in vitro [7–9]. Activation of PPAR*α* has been demonstrated to inhibit inflammatory process; therefore, it may play an important role in the development of restenosis after vascular injury.

Central to the inflammatory process following arterial injury is the rapid upregulation of inflammatory cytokines and coagulation factors. It has been demonstrated that PPAR*α* activators inhibited VCAM-1 expression and synthesis of MCP-1 and reduced monocyte binding to activated human endothelial cells [10, 11]. Furthermore, Kopp et al. demonstrated that the decreased recruitment of monocytes after vascular injury is mediated through inhibition of the tissue factor (TF) pathway [12]. TF is the major physiologic activator of coagulation in vivo and was shown to mediate a prolonged prothrombotic state after balloon angioplasty [13, 14]. TF has also been demonstrated to contribute to restenosis by nonthrombotic mechanisms by eliciting a proinflammatory response [15, 16]. Consistent with these observations, recent in vitro studies showed that PPAR*α* activators inhibit tissue factor expression in human monocytes and macrophage [17, 18]. Based on these findings, this study was undertaken in vivo to test the hypothesis that PPAR*α* activation would decrease monocyte chemoattractant protein-1 (MCP-1), which would decrease leukocyte infiltration into the arterial wall, and TF expression following arterial injury, ultimately leading to decreased neointimal formation.

## 2. Methods

### 2.1. Arterial Injury

Mice (8 to 10 weeks old) with a targeted disruption of the PPAR*α* gene (PPAR*α* ^−/−^) and littermate wild-type controls (WT) were purchased from Jackson Laboratories. Four groups of animals, PPAR*α* ^−/−^ and WT mice, treated or untreated with Wy14643 (10 mg/kg), underwent carotid air-desiccating/pressure double injury. Briefly, the external carotid artery was ligated, and an incision was made in the external carotid artery, and a 30-gauge needle connected to a saline-filled syringe with tubing was introduced. After irrigating the isolated common carotid artery with saline to remove blood, the syringe was replaced with an angioplasty inflation device, and the isolated, saline-filled common carotid segment was dilated at the pressure of 1.5 atmospheres for 30 seconds. The inflation device was replaced with an air-filled 60mL syringe, and a 30-gauge air-exit hole was made at the proximal end of the common carotid artery. Endothelium was injured by air-desiccating the carotid artery for 3 minutes (20 mL/min). After air drying, the artery was refilled with saline, and the needle was removed, and anterograde blood flow was reestablished. The wound was irrigated with saline and closed.

### 2.2. Administration of PPAR*α* Activator

Wy14643 (10 mg/kg) was administered by gavage daily beginning 7 days before injury through the followup. 

### 2.3. Tissue Harvest and Preparation

The animals were sacrificed at 4 and 21 days after injury. For animals sacrificed at 4 days, the excised carotid arteries were snap-frozen in liquid nitrogen and stored at –70°C for later PCR analysis. For animals sacrificed 4 and 21 days after injury, the injured vessel segments were perfusion-fixed with 5% Histochoice (Amresco) for 5 minutes and then harvested. Specimens were stored in 5% Histochoice for at least 24 hours before embedding. 

### 2.4. RNA Purification, Microarray Assay and RT-PCR for MCP-1 and TF mRNA Expression 

Total RNA from vessel segments was extracted from frozen tissue using RNaqueous-Micro Kit (Catalog: 1931, Ambion). Total RNA from each carotid artery was subsequently suspended in 20ul DEPC water. Each carotid artery will yield approximately 50ng of total RNA. Chemiluminescent cDNA probes for microarray were synthesized with GEArray Ampolabelling-LPR kit (Catalog: L-03, Superarray). 9 uL of total RNA will be needed for each reaction. The synthesized probes were then hybridized with array membrane (GEArray Q series Mouse Chemokines and Receptors Gene Array: MM-005) and subsequently processed by using Chemiluminescent detection kit (Catalog D-01, Superarray). The signals were detected with CCD camera per protocol. The same RNA purification protocol was used for RT-PCR of mouse TF and MCP-1. Reverse transcription reaction was conducted with TaqMan gene expression system (Applied Biosystems). Premixed primer sets for MCP-1, TF, and 18sRNA control were ordered from Assay-on-Demand service, Applied Biosystems (mm00441242_m1, mm0038853_m1, HS 99999901_s1). We found that Assay-on-Demand yields more consistent results than Cybergreen based PCR reaction. After the 40th cycle, the PCR products were separated on a 0.8% agarose gel to verify that the appropriate product was obtained, and that no other products were generated. Using this system, the cycle number of the half-maximal signal in each group was obtained. Then the cycle number necessary to obtain a half-maximal signal for GAPDH was used to normalize the cycle number obtained for each sample. 

### 2.5. Morphometry

The fixed carotid arteries were embedded in paraffin and cut in serial sections, at 5 mm apart, from the proximal to distal end. The slides were stained with hematoxylin-eosin (H-E) and Van Gieson Elastic. An observer blinded to the study quantified morphometric analysis using a computerized digital microscopic planimetry software package (Image-Pro Plus, Version 4.0 for Windows). The section from each injured arterial segment exhibiting the most severe degree of luminal narrowing was assessed as the “lesion” point. The neointimal and medial boundaries were determined, the cross-section areas were subtended by the luminal border, the internal elastic lamina (IEL) and the external elastic lamina (EEL) were measured, and the ratio of intimal and medial area (I/M) was calculated.

### 2.6. VSMC Proliferation/DNA Synthesis Assay

For the animals sacrificed 4 days after injury, the effect of PPAR*α* and its activation on VSMC proliferation was evaluated. The mice received intraperitoneal injection of bromodeoxyuridine (BrdU) (2.5 mg/injection, Sigma Chemical Co.) at 18, 12, and 1 hours before euthanasia. The assessment of DNA synthesis in vivo was performed using BrdU In Situ Detection kit (BD Biosciences). On application of biotinylated anti-mouse IgG secondary antibody, the sections were counterstained with hematoxylin. The numbers of BrdU-positive nuclei per section were counted by two observers blinded to the treatment regimens, and the labeling index (positive nuclei/total nuclei) was calculated.

### 2.7. Immunohistochemistry

The inflammatory response 4 days after injury was assessed by immunostaining CD45, a specific marker for leukocytes. CD45-positive cells were immunolocalized by incubation with a mouse monoclonal antibody against CD45 secondary antibody (1.25 *μ*g/mL) for 30 minutes. Detection of CD45 was completed with DAB chromogen substrate that produces a brown cells surface stain on CD45-positive cells. The expression of CD45 was semiquantified by determining the percent-positive area in a blinded manner using the computerized digital microscopic planimetry algorithm as described above. In addition, double immunofluorescence staining was performed to identify the cell source of TF by staining for CD45 and *α*-actin. Briefly, nonspecific binding sites were blocked in blocking buffer containing 1% bovine serum albumin and 1% donkey serum in PBS after deparaffinization and antigen retrieval. Then incubation of antibodies was performed overnight in blocking buffer. The following antibodies were used: rabbit anti-TF antibody (American Diagnostica Inc.), goat anti-*α*-actin of smooth muscle cells antibody (Sigma), and goat anti-CD45 antibody (Santa Cruz Biotechnology Inc.). The quantification was performed by counting the positive-stained cells. 

### 2.8. Statistical Analysis

All data were expressed as mean SD. Statistical analysis was performed with use of SPSS software (Version 10.0 for Windows, SPSS Inc.). Continuous variables were compared using ANOVA analysis with Bonferroni correction. A value of *P* ≤ 0.05 is considered to be statistically significant.

## 3. Results

### 3.1. Effects of PPAR*α* Activation on Neointimal Formation

**Figure 1:**
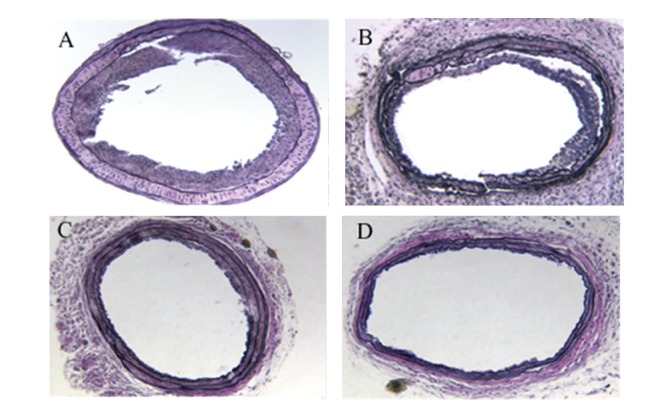
Photomicrographs of mice carotid arteries 21 days after injury (elastic staining, 63X). (A) representative cross-section of injured artery from PPAR*α* knockout mouse; (B) representative cross-section of injured artery from PPAR*α* knockout mouse treated with Wy14643; (C) representative cross-section of injured artery from WT mouse; (D) representative cross-section of injured artery from WT mouse treated with Wy14643.

 Morphometric data from samples 21 days after injury as shown in Table 1 demonstrated that (1) WT type mice have larger luminal areas as compared with PPAR*α* ^−/−^ mice. (2) Significantly more neointimal formation was found in PPAR*α* ^−/−^ mice compared with WT mice (0.020 ± 0.009 mm^2^ versus 0.009 ± 0.005 mm^2^, *P* < 0.05). (3) The activation of PPAR*α* with its activator Wy14643 resulted in a further decrease in neointimal formation when compared to untreated WT mice (0.005 ± 0.003 versus 0.009 ± 0.005 mm^2^, *P* < 0.05). (4) This significant difference of neointimal formation still existed when normalized to media area (neointima/media ratio: 0.610 ± 0.244 from PPAR*α* ^−/−^ group versus 0.379 ± 0.231 from WT group and 0.204 ± 0.116 from WT treated with Wy14643 group, *P* < 0.05). However, no beneficial effects were observed in the PPAR*α* knockout mice treated with PPAR*α* activation. (5) No difference was found in EEL (external elastic lamina) comparing the WT and PPAR*α* knockout animals (Figure 1). 

### 3.2. Effect of PPAR*α* on Cell Proliferation

**Figure 2:**
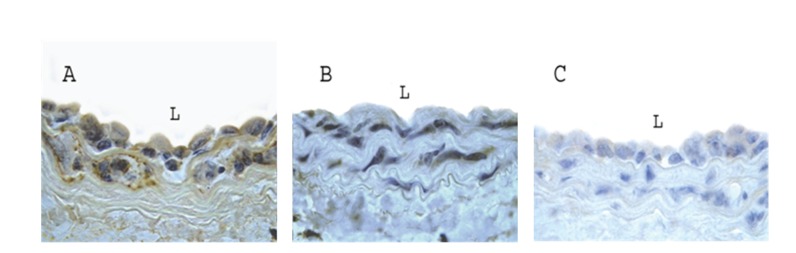
Representative pictures of BrdU-positive cells in carotid arteries from PPAR*α* knockout mouse, WT mouse, and WT mouse treated with Wy14643 at day 4 after injury (100x). (A) PPAR*α* knockout group; (B) WT group; (C) WT group treated with Wy14643. BrdU-positive cells were much more in the PPAR*α* knockout group when compared to WT group. PPAR*α* activation further decreased BrdU-positive cells.

Four days after injury, the number of BrdU-positive cells in the intima and media was much less in the WT group when compared to PPAR*α* ^−/−^ group (29 ± 12 versus 58 ± 11 cells per cross section; *P* < 0.05), and the number of BrdU-positive cells was further decreased by treatment with Wy14643 (8 ± 3 cells per cross section, *P* < 0.05 when compared to PPAR*α* ^−/−^ and WT groups, resp.) (Figure 2), indicating that Wy14643 significantly suppressed VSMC proliferation after injury.

### 3.3. Assay of Leukocyte Infiltration

**Figure 3:**
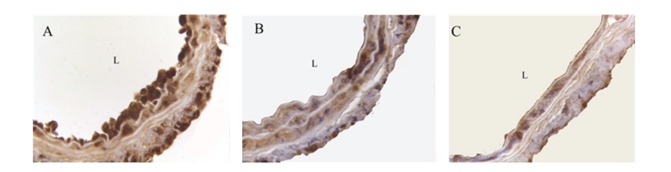
Photomicrographs of CD45 immunostaining showing injured carotid arteries from PPAR*α* knockout mouse, WT mouse, and WT mouse treated with Wy14643 at day 4 after injury (63x). (A) Representative cross-section of CD45 expression from PPAR*α* knockout group. (B) Representative cross-section of CD45 expression from WT group. (C) Representative cross-section of CD45 expression from WT treated with Wy14643 group. More extensive CD45 expression was observed in PPAR*α* knockout mouse when compared to WT mouse. The treatment with Wy14643 further decreased CD45 expression.

To characterize the cell proliferation and recruitment in the injured, site, immunohistochemical analysis was performed by using antibody against CD45. PPAR*α* ^−/−^ group showed more extensive CD45 expression when compared to WT group, as assessed by percent-positive area for CD45-positive cells (16.31% ± 3.25 versus 9.87% ± 2.62, *P* < 0.05). The treatment with Wy14643 decreased CD45 expression (5.09% ± 2.15%, *P* < 0.05 when compared to PPAR*α* ^−/−^ and WT without Wy14643 treatment groups, resp.) (Figure 3). 

### 3.4. Chemokine and Tissue Factor Expression

**Figure 4:**
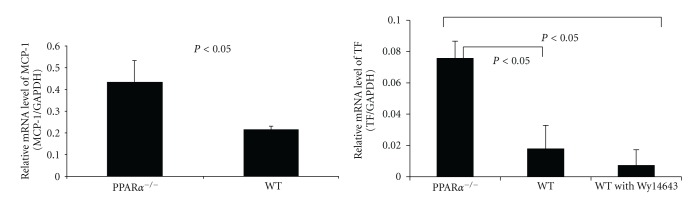
The effects of PPAR*α* and PPAR*α* activation on MCP-1 and TF expression after arterial injury determined by real-time PCR. (a) MCP-1 mRNA level was much higher in the PPAR*α* knockout group when compared to WT group; (b) TF mRNA level was much higher in the PPAR*α* knockout group when compared to WT group and WT group treated with Wy14643. PPAR*α* activation further decreased TF expression when compared to untreated WT group but did not reach statistic significance.

Gene array of mouse inflammatory cytokines and their receptors was used to analyze the gene profiling of individual injured carotid artery from WT and PPAR*α* ^−/−^ animals 4 days after injury. The changes in expression of 67 chemokine genes were compared in WT and PPAR*α* ^−/−^ mice. MCP-1 mRNA was consistently found to be increased in injured artery from PPAR*α* knockout animals (data no shown). The changes observed in MCP-1 expression were confirmed by real-time PCR (RT-PCR) in individual vessels (*n* = 4) 4 days after injury and summarized in Figure 4(a). MCP-1 levels were upregulated in PPAR*α* ^−/−^ mice compared with WT mice (MCP-1: 0.4319 ± 0.099 versus 0.2149 ± 0.014;  *P* < 0.05, unit = MCP-1/GAPDH, 2-fold increase). The expression of other chemokines was not observed to be significantly different between WT and PPAR*α* ^−/−^ mice 4 days after arterial injury.

Tissue factor expression has been linked to neointimal hyperplasia; therefore, we quantified TF expression 4 days after arterial injury. We observed significantly greater TF expression in the PPAR*α* ^−/−^ mice compared to WT mice (TF: 0.0756 ± 0.011 versus 0.0178 ± 0.015, *P* < 0.05, unit = TF/GAPDH, 4-fold increase). However, PPAR*α* activation did not further downregulate TF expression in the WT mice treated with Wy14643 group (0.0072 ± 0.01), (*P* < 0.05, unit = TF/GAPDH) (Figure 4(b)).

### 3.5. Immunohistochemical Assay

**Figure 5:**
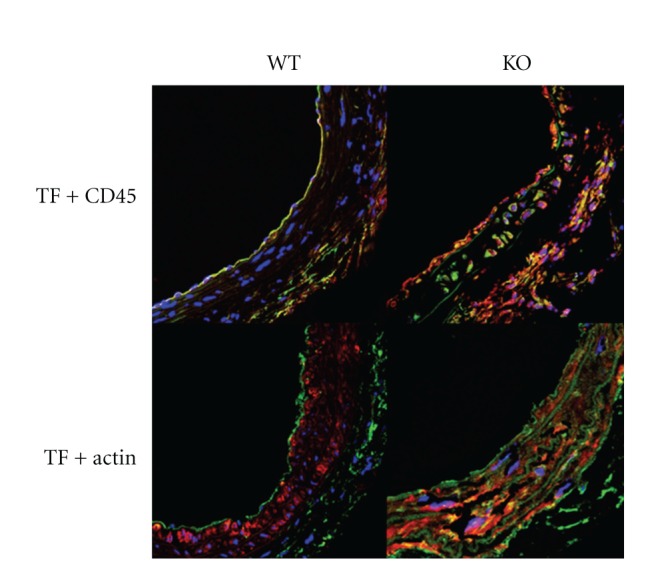
Double immunofluorescent staining for identifying the cell source of TF. Optical sectioning was averaged over four frames and the image size was set at 1024 × 1024  pixels. There were no digital adjustments made to the images. TF with CD45: TF (Green), CD45 (Red) & DAPI (Blue). TF with SMC *α*-actin: TF (Green), *α*-actin (Red) & DAPI (Blue). Our data indicated that more TF-positive cells were found in the PPAR*α* ^−/−^ mice, which is overlapped with the CD45-posttive cells, suggesting CD45-positive cells as the source of TF.

To determine the cell source the TF, double immunofluorescence staining was done by using antibodies against CD45, SMC *α*-actin, and TF on injured arteries. It is shown that more TF-positive cells were found in the PPAR*α* ^−/−^ mice. TF-positive signals are mainly overlapped with the CD45-positive cells, and to lesser extent with *α*-actin staining in PPAR*α* ^−/−^ mice (Figure 5), indicating CD45-positive cells as the major source of TF 4 days after arterial injury. In the WT animals, SMC did not contribute to TF expression 4 days after the injury.

## 4. Discussion

The PPAR regulatory pathway plays a critical role in the regulation of diverse biologic processes within the cardiovascular system. Inflammatory response caused by vascular injury has been recognized as a pivotal player in the development of atherosclerosis and restenosis. Although much effort has been devoted to effective therapy, effective prevention measure has not been developed. The aim of the present study was to examine the effects of PPAR*α* ^−/−^ in an experimental model of arterial injury and highlights this receptor's potential role in the prevention of stenosis. We demonstrate for the first time in vivo that the absence of the PPAR*α* gene significantly increases (i) the neointima formation, (ii) the infiltration of CD45-positive cells, (iii) the expression of MCP-1, and (iv) the expression of TF in CD45-positive cells. Our study is also the first study using microarray technique to specifically analyze the expression of chemokines and their receptors by using mice carotid artery. 

PPAR*α* is expressed mainly in the liver, kidney, and skeletal. However, recent study indicates that PPAR*α* is expressed at substantial levels in vascular cells including endothelial cells, monocyte/macrophages, and VSMCs where it has been shown to function as a negative regulator of proinflammatory processes [5]. The damage of endothelial cells, recruitment of monocytes, SMC proliferation, and thrombus formation are involved in the response to vascular injury. Therefore, PPAR*α* actively involved the complicated array of gene expression in these cell groups. Staels et al. showed that PPAR*α* is actively involved in the regulation of inflammatory cytokines expression [9]. Anti-inflammatory action of PPAR*α* activators is mediated by interfering with the NF-*κ*B and AP-1 pathways [8, 9]. Furthermore, the correlation of PPAR*α* with inflammation is corroborated by the demonstration that PPAR*α* activators inhibited VCAM-1 expression and synthesis of MCP-1 and reduced monocyte binding to activated human endothelial cells [10, 11]. PPAR-*α* agonists have been shown to inhibit inflammation in VSMCs, and conversely PPAR-*α* deficiency leads to more profound inflammation in these cells [19, 20]. In the current study, we demonstrated that leukocyte recruitment and MCP-1 expression following arterial injury were decreased in animals treated with PPAR-*α* activator and increased in PPAR-*α* ^−/−^ mice, indicating that anti-inflammatory effect of PPAR-*α* activation is through inhibition of leukocyte recruitment leading to the downregulation of TF expression in both CD45 positive and SMC cells. Consistent with these observations, in the current study, we demonstrated that leukocyte recruitment and MCP-1 expression following arterial injury were decreased in animals treated with PPAR*α* activator and increased in PPAR-*α* ^−/−^ mice.

Procoagulant mechanism is thought to contribute significantly to the initiation of restenosis. TF is the primary initiator of the coagulation cascade and is thought to play a key role in the generation of arterial thrombosis [14, 17]. Recent studies have suggested that TF mediates inflammatory processes in the arterial wall and may be an important regulator of intimal hyperplasia [12, 21, 22]. Giesen et al. demonstrated that TF activity in the media was increased as early as 24 hours after injury and subsequently accumulated in the neointima [23]. Kopp et al. demonstrated that inhibition of TF pathway significantly reduced restenosis by suppressing the recruitment of monocytes and MCP-1 expression after balloon injury [12]. More recently, decreased intimal hyperplasia was observed in the low-TF mice [24]. Our study demonstrates for the first time in vivo that PPAR*α* plays an important role in TF expression in both CD45-positive cells and SMC, and its activation results in the interruption of the process of neointimal formation after vascular injury.

Our double immunostaining showed the CD45-positive leukocytes as the major cell source of TF, indicating that anti-inflammatory effect of PPAR*α* activation is through inhibition of leukocyte recruitment leading to the downregulation of TF expression in both CD45-positive and SMC cells. Recently, PPAR*α* agonists have been shown to downregulate TF expression in human monocytes and macrophage [18, 25, 26], and PPAR*α* agonists inhibit activity of the TF promoter as well as TF protein levels in macrophages, which further supports our finding from in vivo model system. 

There is no detailed study of TF promoter for PPAR*α* response element, (PPREs) which are typically organized as direct repeats of the core sequence AGGTCA separated by 1 or 2 nucleotides (DR1 and DR2), flanked upstream by A/T-rich sequences. However, further treatment with Wy14643 did not suppress TF expression in our study. One possibility is that TF expression is differentially regulated in monocytes and smooth muscle cells. Oxidized LDL has been shown to modulate TF expression in SMC via SP-1 and Egr-1, whereas lipopolysaccharide induction of TF in monocytes is via AP-1 and NFkB [27, 28]. Thus, based on our results, we propose that PPAR*α* modulates TF expression by inhibiting leukocyte recruitment that follows arterial injury. In WT mice, MCP-1 expression is low; therefore, Wy14643 does not significantly alter TF expression, because PPAR*α* does not directly modulate TF expression in SMC. 

In conclusion, PPAR*α* plays an important role in significant decrease of neointimal formation by decreasing MCP-1 expression leading to decreased leukocyte recruitment and tissue factor expression in the injured artery.

## Figures and Tables

**Table 1:** Morphometric data at 21 days.

	PPAR*α* ^−/−^ (*n* = 11)	WT (*n* = 13)	WT with Wy14643 (*n* = 15)	PPAR*α* ^−/−^ with Wy14643 (*n* = 10)
Luminal area (mm^2^)	0.068 ± 0.019	0.088 ± 0.018	0.086 ± 0.021	0.069 ± 0.014
Neointimal area (IA, mm^2^)	0.020 ± 0.009^∗^	0.009 ± 0.005^§^	0.005 ± 0.003	0.021 ± 0.007^∗^
Medial area (MA, mm^2^)	0.033 ± 0.004	0.026 ± 0.011	0.030 ± 0.017	0.030 ± 0.012
EEL area (mm^2^)	0.122 ± 0.024	0.123 ± 0.026	0.122 ± 0.031	0.128 ± 0.021
IA/MA	0.610 ± 0.244^∗^	0.379 ± 0.231^§^	0.204 ± 0.116	0.798 ± 0.219^∗^

EEL: external elastic lamina.

*P < 0.05 when compared to both WT without and WT with Wy14643 groups.

^§^ *P* < 0.05 when compared to both PPAR*α* ^−/−^ and WT with Wy14643 groups.
